# Stable clinical long term results after AMIC in the aligned knee

**DOI:** 10.1007/s00402-020-03564-7

**Published:** 2020-08-13

**Authors:** Nadine Kaiser, Roland P. Jakob, Geert Pagenstert, Moritz Tannast, Daniel Petek

**Affiliations:** 1 University Hospital of Berne, Freiburgstrasse 15, 3010 Bern, Switzerland; 2 Kantonsspital Fribourg, Chemin Des Pensionnats 2-6, 1700 Fribourg, Switzerland; 3 CLARAHOF Orthopaedics, Clarahofweg 19a, 4058 Basel, Switzerland

**Keywords:** Autologous matrix-induced chondrogenesis, AMIC, Bone marrow stimulation, HTO, Patellofemoral maltracking

## Abstract

**Introduction:**

The aim of this study was to report a long-term follow-up of patients treated with autologous matrix-induced chondrogenesis (AMIC) for full-thickness chondral and osteochondral defects of the femoral condyle or patella combined with the correction of lower limb malalignment or patellar tracking if indicated.

**Methods:**

Thirty-three patients (thirty-four knees) were treated surgically for chondral and osteochondral cartilage defects of the knee joint. Regarding the origin of the lesion, patients were divided into three groups. Chondral lesions were observed in the patella (cP group) in fifteen patients, whereas eight patients demonstrated a femoral condylar location (cF group). Eleven patients presented with osteochondritis dissecans of the femur (ocF group). Associated procedures involving realignment of the patella, osteotomy around the knee, or cancellous bone grafting were performed when necessary. The mean size of the lesions was 2.8 ± 1.6 cm^2^, and the mean patient age was 37.1 ± 11.9 years. To evaluate the clinical outcomes, the Lysholm score and the VAS pain score were imposed, as well as the reoperation rate.

**Results:**

After an average of 9.3 ± 1 years, follow-up was completed in 79% of the patients. Two patients from the cohort received a total knee prosthesis. The primary outcome measures (Lysolm and VAS pain) at 9-year follow-up were 85 ± 13 for the Lysholm score and 1.9 ± 1.6 for the VAS score in the entire analyzed population. Compared to the preoperative values (Lysholm 56 ± 19, VAS 5.8 ± 2.4) and the 2-year results (Lysholm 85 ± 16, VAS 2.0 ± 2.1), there was significant improvement in the first 2 years after intervention and a stable course in the long-term observation. The same was observed in the cP and ocF subgroups, whereas patients of the cF group showed even further improvement.

**Conclusions:**

AMIC showed durable results in aligned knees. The favorable outcome was maintained after an average of 9 years when malalignment of the lower limb and patellar maltracking were corrected. Such data are particularly encouraging for young adult patients who may benefit from a procedure that circumvents early arthroplasty.

## Introduction

Articular cartilage in the adult is a type of highly specialized connective tissue that is built to last a lifetime. The half-life of type II collagen is estimated to be approximately 117 years [1], which is an underlying reason why its regenerative potential is limited. Mature chondrocytes, imbedded in a healthy structural framework of collagen, have low anabolic and proliferative activities and therefore a limited need for vascular support or nerve supply. Because of this, there is very limited healing potential for cartilage defects in cases of damage. The consequence of this inadequate repair leads to irreversible cartilage degeneration and causes osteoarthritis [2].

Therefore, partial-thickness cartilage tears will not heal once formed, whereas full-thickness osteochondral defects will partially fill with fibrocartilage scar tissue. The concept of using the intrinsic healing potential of the full-thickness cartilage lesion was first described by Pridie in 1959 and was further developed by Steadman to the use of microfracture (MFx), and this concept is based on the fact that mesenchymal stem cells (MSCs) arising from subchondral bone marrow will migrate toward the defect [3, 4]. A further evolution of MFx has been described by Behrens et al., who published their results on the use of autologous matrix-induced chondrogenesis (AMIC) [5]. In this technique, the blood clot that forms in the defect after MFx is stabilized by a collagen I/III membrane (Chondro-Gide^®^, Geistlich Pharma AG, Switzerland), providing an additional framework and more stability against shear forces in joint motion [6]. Since its introduction, AMIC has shown good short- and mid-term results [7, 8] that are comparable to those of MFx [9]. More recent data, based on a randomized controlled clinical trial, have analyzed the 5-year results after AMIC procedures in the knee versus microfracture alone [10]. These data showed significant clinical improvement for the first 2 years in both the microfracture and AMIC groups. Thereafter, a progressive and significant degradation in the functional score was observed in the MFx group by 5 years, while all functional parameters remained stable for at least 5 years of follow-up in the AMIC group. Additionally, stable results after 5 years have recently been published for the AMIC technique in the knee [11] and the hip, as well as for talar cartilage repair [12, 13].

Currently, it is well accepted that in the case of compartmental overload, a lower limb alignment correction is mandatory in combination with cartilage repair for medial or lateral compartment cartilage defects [14, 15]. Realignment of any extensor mechanism maltracking is also a needed concomitant procedure in patellofemoral joint lesions [16, 17] to optimize biomechanics in the knee and influence clinical outcome.

The purpose of this 10-year study is to retrospectively analyze the clinical outcomes of AMIC procedures. The hypothesis is that the promising results of the AMIC procedure will remain good, especially when alignment is maintained.

## Materials and methods

All patients treated with an AMIC procedure in the HFR-Fribourg District Hospital (Switzerland) for full-thickness cartilage tears of the knee between 2003 and 2006 were retrospectively reviewed. All patient data have been fully anonymized and collected according to institutional board recommendations.

The inclusion criteria were as follows: full documentation of the clinical examination, presence of preoperative Lysholm score, and VAS pain score; full documentation of the defect localization and size; and a complete postoperative follow-up at 2 years that included the Lysolm score and VAS pain score. All patients with complete documentation of the above were included to the final follow-up.

Thirty-three patients (34 knees) who met the inclusion criteria were identified. Patients completed the scores either during a standard visit at our clinic or where contacted by phone. According to the etiology and location of the chondral lesion, the patients were divided into three groups. All concomitant procedures performed in patients who received AMIC were also reported. The first group consisted of fifteen patients with a chondral lesion of the patella (cP group). The second group presents eight patients with pure chondral defects in one of the femoral condyles (cF group). The third group includes ten patients (eleven knees, one bilateral case) with osteochondral lesions of the femur (ocF group). All lesions were in a chronic status; there were no acute traumatic lesions. There was no difference between the baseline criteria of all patients within the 3 groups (i.e., age and defect size), except for the localization and associated treatment procedures. The baseline demographics of all groups are shown in Table 1.

**Table 1:** Baseline data of all 33 patients (34 knees)

	All	cP	cF	ocF
Number	33 (34)	15	8	10 (11)
Age (years)	37.1 ± 11.9	40.2 ± 12.9	38.0 ± 9.9	32.2 ± 11.0
Sex	22m/11 f	8m/7f	7m/1f	7m/3f
Localisation				
Patella	15	15	–	–
Medial femoral condyle	14	–	7	7
Lateral femoral condyle	4	–	1	3
Femoral trochlea central	1	–	–	1
Outerbridge				
2°–3°	2	1	3	0
3°	5	0	5	0
4°	3	5	18	10
Unknown	5	2	8	1
Defect size (cm^2^)	2.8 ± 1.6	2.6 ± 0.9	2.9 ± 2.3	3.0 ± 1.6
Concomitant treatment				
Tibial osteotomy	11	–	7	4
Femoral osteotomy	2	1	–	1
Patella realignment	1	15	1	–
Cancellous bone graft	9	–	–	9
Meniscal repair	5	3	–	2
Ligament repair	1	–	1	-

Subgroups ocF: cP: chondral lesion of the patella; cF: chondral femoral lesion; osteochondral femoral lesion

### Indication

As the indication for AMIC cartilage repair is highly related to axial alignment, the senior author performed a thorough analysis of any patellofemoral maltracking or any lower leg axial deviation. One-leg standing films (coronal, 45° coronal, lateral, and patellar axial) were routinely performed as well as long-leg views. In addition, MRI or CT scans were obtained when necessary. If malalignment of the lower extremity was identified (varus or valgus deviation), the decision of leg axis correction was made and planned as a concomitant surgery during the AMIC procedure. At that time, any deformity above 5° of varus or 3° of valgus was considered as a malalignment and therefore corrected. The amount of correction aimed to reach 3° of valgus in varus knees and a neutral alignment for valgus knees. Patellofemoral maltracking when associated with recurrent patellar dislocation or pathological TTTG (< 20 mm) led to realignment procedure essentially by a transfer of the tibial tubercle, a lateral release, and a reinforcement of the vastus medialis muscle. Because of this policy, knees during the performed cartilage procedures were not subjected to any mechanical overload.

**Fig. 1:**
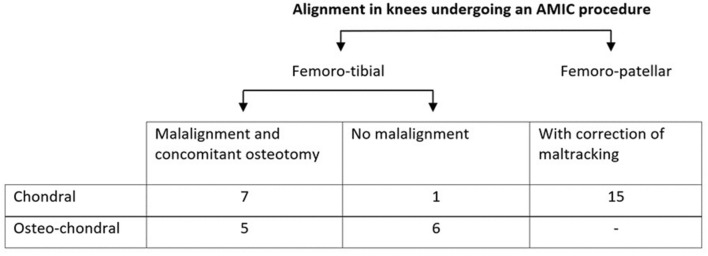
Summary of concomitant alignment procedures in 33 patients (34 knees)

All of the patients in the cP group received realignment of the extensor mechanism during the same surgery. In the cF group, axial corrective osteotomy was performed in seven patients (coronal tibial correction in six patients and sagittal femoral correction in one patient) in conjunction with cartilage repair. In the ocF group, five coronal osteotomies (four tibial valgizations and one femoral varization) were associated with the AMIC procedure. Among these ocF patients, nine received a cancellous bone graft as well because the sub chondral bone stock was considered to be insufficient after debridement (Fig. 1).

### Surgical procedure

First, arthroscopy was performed to confirm the location and size of the defect as well as the feasibility of the AMIC procedure. This first step was followed by lower limb osteotomy when indicated.

**Fig. 2:**
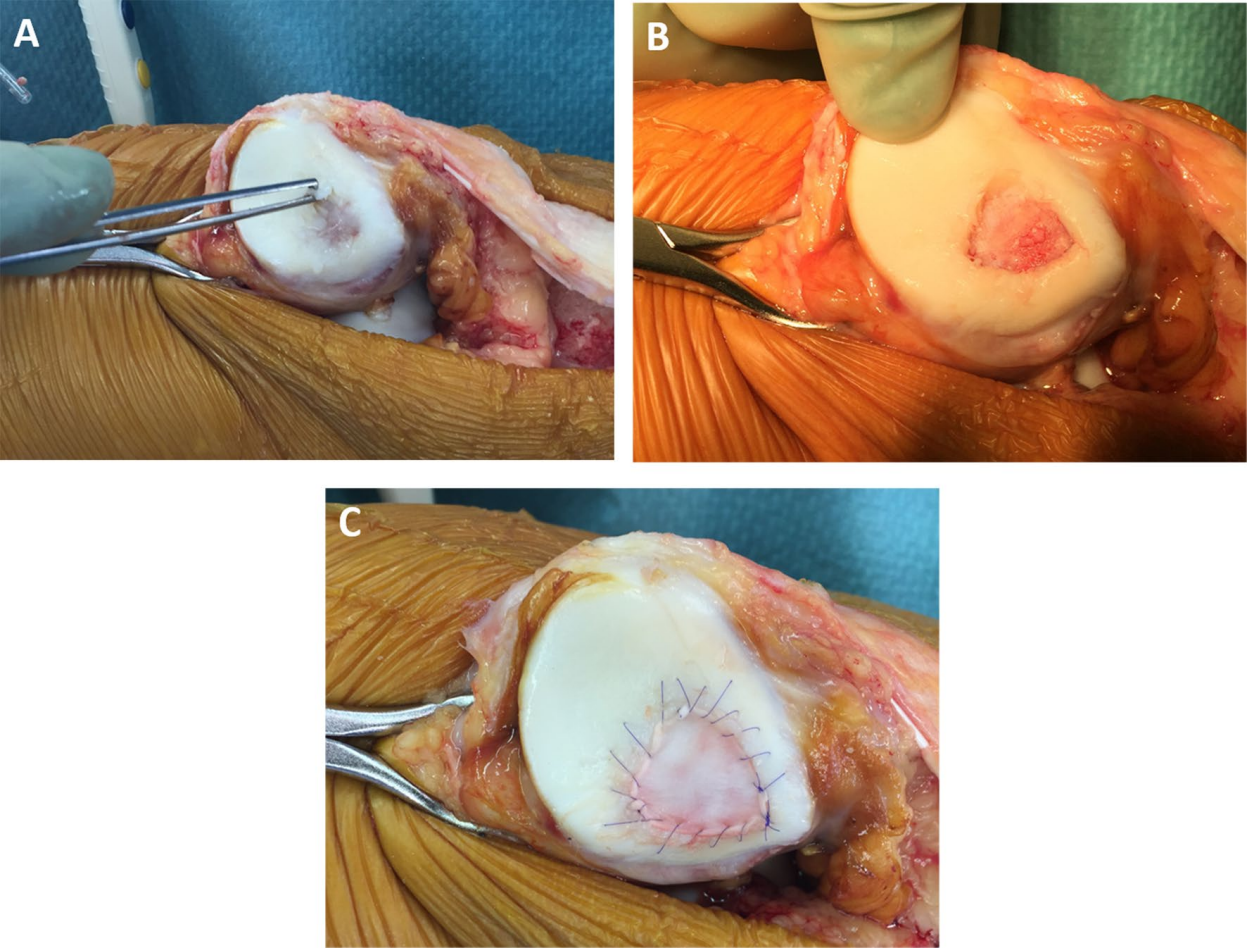
**a**–**c** Intraoperative images of an AMIC procedure for a retropatellar chondral defect. **a** Initial defect; **b** Pridie drilled surface; **c** defect covered with a sutured collagen I/III membrane

Then, an open procedure that consisted of debridement and excision of the loosened cartilage fragment followed by Pridie drilling of the sclerotic bone and coverage of the defect with a collagen I/III membrane (Chondro-Gide^®^, Geistlich Pharma AG, Switzerland) was performed under a tourniquet. In the first six patients, the membrane was sutured to the surrounding healthy cartilage only. For the subsequent patients, the membrane was sutured and glued with Tissucol^®^ (Baxter, Unterschleissheim, Germany). For the chondral defects close to the cartilage margins, the membrane has been sutured to the adjacent periosteum. At the end of the procedure, the tourniquet was released, and the correct filling of the defect by blood clotting was confirmed (Fig. 2).

### Outcome measures

The primary endpoints were the clinical Lysholm and VAS scores reported by the patients. All patients completed a questionnaire for the Lysholm score [18] and the VAS score for the assessment of preoperative pain, and they also completed these questionnaires at the 2- and 10-year follow-up assessments. The Lysholm score was used as the primary outcome score because the Lysholm score is reliable and valid in this setting [18], and it has been previously used by several authors for similar indications [8, 16, 18–21]. The secondary endpoint was revision surgery or reoperation during the 10-year follow-up period.

### Statistical analysis

Qualitative variables were described by absolute and relative frequencies of their categories. This analysis was performed per group and for the pooled data. Distributions of numeric variables were summarized by the number of non-missing values, means, standard deviations, extreme values, and three quartiles. This analysis was assessed per group and for the pooled data. The study groups were compared by approximate Kruskal–Wallis tests regarding numeric variables and by exact Fisher–Freeman–Halton tests in the case of categorical variables. Within a study group and pooled over all study groups, pairwise exact two-sided Wilcoxon signed-rank tests were used to assess significance over time. The presented *p* values are Bonferroni corrected, i.e., multiplied by three because there were three tests (0 vs 2, 0 vs 10, and 2 vs 10). All tests were performed in an exploratory manner with a 5% level of significance.

All statistical analyses were performed using the statistics software R version 3.3.2 along with package “coin” for exact rank tests [22].

As this was a retrospective, non-comparative study, power calculation was not performed. The sample size was determined by the number of cases that were recruited. This procedure has already been described in a respective paragraph of the STROBE explanatory article [23].

## Results

**Fig. 3:**
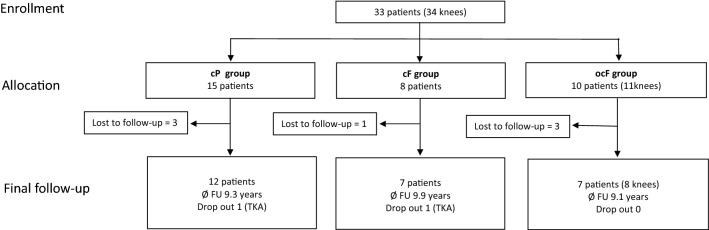
CONSORT flow diagram showing the progression of all 33 patients enrolled in this study. Subgroups: cP: chondral patella group; cF: chondral femoral group; ocF: osteochondral femoral group; All patients completed the 2-year follow-up. There were 7 patients lost to follow-up between 2- and 9-year control

We included 33 patients (34 knees). Seven patients were lost to follow-up.. The follow-up rate of the cohort was therefore 26/33 patients (79%). From the remaining 26 patients, two underwent revision surgery: a total knee prosthesis for degenerative changes was implemented 9 and 10 years after the AMIC procedure. The first patient belonged to the cF group, and the second patient belonged to the cP group. As clinical scores of these patients are not referable to the AMIC or initial procedure, these patients were excluded from further interpretation (two drop out). The final follow-up was completed in 24 patients (25 knees). The mean time to follow-up was 9.3 ± 1.0 years. Details of patient flow are shown in Fig. 3.

**Fig. 4:**
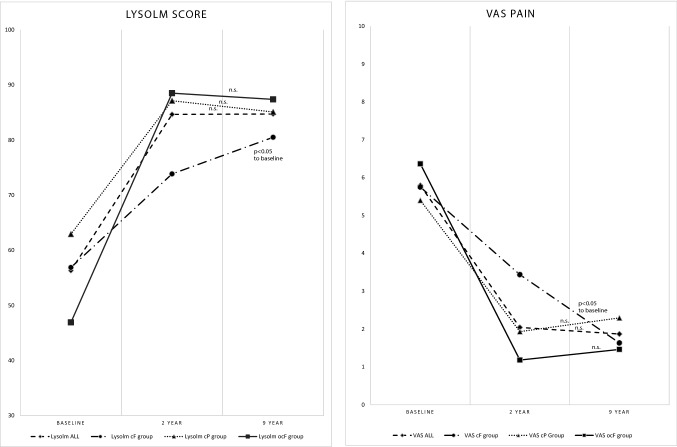
Mean Lysholm score and VAS pain scale pre-operatively and 2 and 9 years after AMIC

At the 9-year follow-up, the primary outcomes (Lysholm score and VAS pain score) for all recruited patients were 85 ± 13 for the Lysholm score and 1.9 ± 1.6 for the VAS score. The values that were measured preoperatively and at the 2-year follow-up showed that there was a significant improvement in the first 2 years. After that, no significant differences were observed between the 2- and 9-year results for both the Lysholm and VAS scores (Table 2, Fig. 4) in the analyzed population.

**Table 2:** Lysholm score and VAS pain scale for all patients and subgroups

Group	Score	Pre-OP	2-year follow up	9-year follow-up	*p* value 2 years vs. pre	*p *value 2 years vs. 9 years	*p* value 9 years vs. pre
All	Lysholm	56 ± 19	85 ± 16	85 ± 13	< 0.001	n.s	< 0.001
	VAS	5.8 ± 2.4	2.0 ± 2.1	1.9 ± 1.6	< 0.001	n.s	< 0.001
cP	Lysholm	63 ± 17	87 ± 14	85 ± 14	< 0.001	n.s	0.005
	VAS	5.4 ± 2.1	1.9 ± 1.8	2.3 ± 2.1	< 0.001	n.s	0.003
cF	Lysholm	57 ± 24	74 ± 19	81 ± 16	n.s	n.s	0.044
	VAS	5.8 ± 3.3	3.4 ± 3.2	1.6 ± 1.2	n.s	n.s	0.013
ocF	Lysholm	47 ± 15	89 ± 14	87 ± 9	n.s	n.s	0.008
	VAS	6.4 ± 2.1	1.2 ± 1.0	1.5 ± 1.1	< 0.001	n.s	0.008

Data presented as mean values ± standard deviation

*cP* chondral lesion of the patella, *cF* chondral femoral lesion, *ocF* osteochondral femoral lesion

In the subgroups, the same, stable course over time was observed in the chondral patellar group (cP) and the osteochondral femoral group (ocF). A different course was observed in the chondral femoral group (cF): in contrast to the other two groups, we found no significant improvement in the first two postoperative years but improvement in the following years compared to the preoperative values, with the best outcome found for this group 9 years after surgery (Table 2, Fig. 4).

**Fig. 5:**
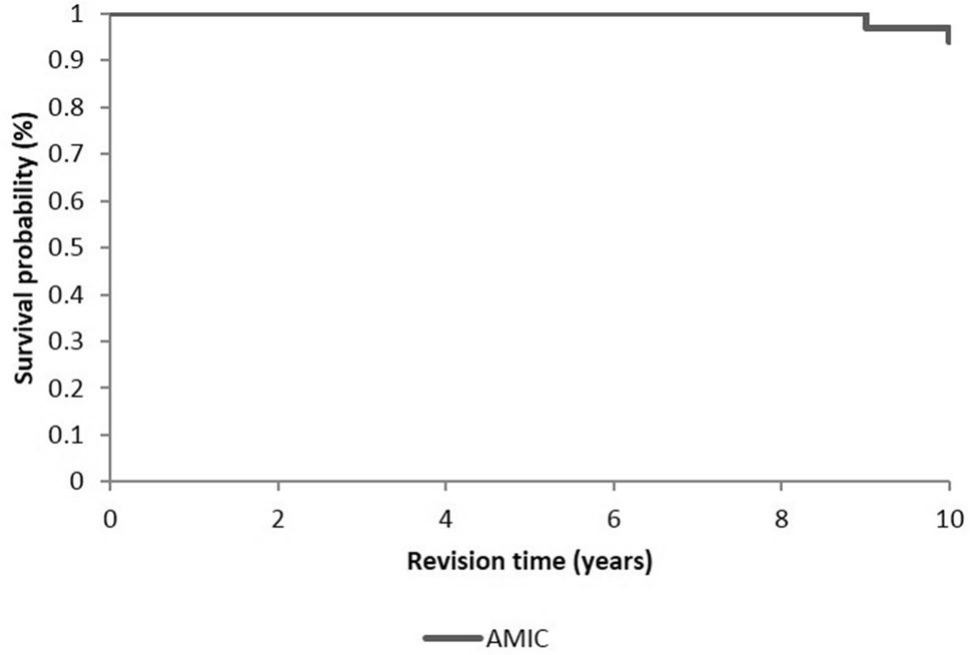
Kaplan–Meier survival curve with total knee arthroplasty (TKA) as the endpoint

The global survival rate in the long-term follow-up in this cohort is expressed by the Kaplan–Meier curve (Fig. 5). Among 26 patients who reached the 10-year follow-up, two patients (7%) received a total knee prosthesis for symptomatic knee arthritis at 9 and 10 years after the initial AMIC procedure.

## Discussion

The most important finding of the present study was that the clinical results of autologous matrix-induced chondrogenesis remained stable up to 10 years after surgery. The presented data show the results of AMIC combined with concomitant realignment procedures whenever needed according to preoperative radiological data. Compared to the clinical outcomes 2 years after surgery, the scores show at least similar results. In the group treated for femoral chondral lesions, there was an even further improvement at 9 years compared to the 2-year results. To the best of our knowledge, this is the first study investigating long-term results of up to 9 years after an AMIC procedure for the knee joint.

These results confirm those of previous investigations regarding AMIC after 2 and 5 years [8, 10, 24] that reported no progressive deterioration, as was observed in the MFx procedure. A newer report from Schiavone Panni et al. also showed promising results with significant clinical and functional improvements for up to 7 years in patients with cartilage defects (> 2 cm^2^) of the knee treated with an AMIC procedure [11]. The presented long-term results support the treatment algorithm proposed by several authors suggesting that for medium-sized defects (2–4 cm^2^), cartilage lesions should be treated by “enhanced microfracture” or “microfracture plus”, which includes protection of the superclot by a covering membrane, corresponding to the AMIC technique [25–27].

These data confirm the hypothesis that clinical benefits may be more robust in AMIC-treated patients. Compared with the results found for patients treated with MFx only, the results of MFx “failures” characterized by revision surgery with either ACI or TKA have been reported in up to 54% of patients 9.8 years after initial procedure [28]. Additionally, a mean value has been established among several MFx studies that reported failures of over 20% 5–10 years after surgery [29, 30]; these presented results seem favorable compared to these data since the revision rate was 7% at 9 years.

Comparing AMIC with autologous chondrocyte transplantation (ACI), a cell-based technic with widespread use for cartilage defects > 2 cm^2^ [31], AMIC has the advantage of being a simple, safe and cost-effective one-step procedure [24]. Regarding long-term results of ACI, there is a durable benefit for the patient in the long-term follow-up [21, 32]. The reported results are comparable to those reported for AMIC [33, 34] but with the burden of the need for a second intervention. Further randomized controlled long-term trials are needed to compare these technics.

Even if these results are promising, some limitations must be considered. First, the heterogeneous patient population with respect to the localization and cause of the underlying lesion, as well as the concomitant procedures performed during the AMIC procedure, must be recognized. The concomitant surgeries during AMIC may confound the results. Therefore, it is not correct to assign the results to the cartilage treatment alone but to the whole treatment strategy decided individually for each patient. The data reported here also reflect a concrete and empiric use of a technique with therapeutic paradigms. At least, and except in some specific traumatic lesions, cartilage defects result from impairments in balance within the knee joint with consecutive focal articular overload. Without adequate treatment of the underlying pathology, there will be no durable improvement of articular knee function. Therefore, outcomes of knee joint articular defects cannot rely only on cartilage therapy because these defects are only one part of the mechanical, biological, and metabolic aspects of knee degeneration. To minimize the bias caused by this fact, we divided the patients into three groups according to the location of the defect, leading to the previously described concomitant procedures.

A common cause of femoro-tibial overload is malalignment of the lower extremity. In our population, eight out of nine patients in the cF group received high tibial osteotomy (HTO). Whether HTO, with or without a concomitant cartilage procedure, can induce or improve cartilage healing is a topic of ongoing discussion. While Kahlenberg et al. with the use of a systematic review showed that HTO and cartilage restoration procedures provided effective and reliable improvements in knee function [19], a recent systematic review published by Filardo et al. concluded that there was no evidence available to support the effectiveness of combined cartilage treatment [35]. Bode et al. compared 19 patients treated with ACI and HTO and 24 patients treated with HTO alone for chondral defects of the knee with coronal malalignment of less than 5°. Even with this minor degree of malalignment, they were able to show a lower rate of reintervention in the combined group compared to the isolated procedure up to 6 years after surgery [14]. A report from Ferruzzi et al. with a follow-up of 11 years compared HTO alone and HTO with ACI as well as HTO with MFx in 56 patients [36]. At the time of the final follow-up, improvements in the clinical and radiographic results were obtained in all patients. HTO associated with ACI showed significantly higher scores than HTO combined with MFx. Interestingly, as already reported for MFx as an isolated procedure, the scores of HTO with MFx showed higher rates of deterioration and progression of osteoarthritis over time. In the group reported here of AMIC associated with HTO, stable clinical results over a period of 10 years after surgery were observed. This confirms the hypothesis that the AMIC procedure combined with the realignment procedure contributes to favorable and stable clinical results.

Regarding axial alignment of lower extremity, there are several reports supporting the correction of patellofemoral maltracking in combination with cartilage repair. Gigante et al. investigated twelve patients with an Outerbridge III° and IV° lesion and patella maltracking defined by a TT-TG > 20 mm. They showed improvements in all clinical scores (Kujala, Tegner, Lysholm, and Cincinnati scores) 36 months after MACI and realignment procedures [16]. A report from Gillogly et al. described 27 patients undergoing an ACI procedure combined with tibial tubercle transfer. Their results showed a significant improvement in symptoms and function in patients with isolated symptomatic patellar chondral defects up to 7 years after surgery [17]. In the present evaluation, all 15 patients with a chondral cartilage lesion (cP group) received a combined procedure, including AMIC and patellar realignment surgery. Similar to the results found in the other groups, a significant functional improvement was measured within the first 2 years and remained stable up to 10 years after surgery.

In the ocF group, nine out of eleven patients received a cancellous bone graft. As published by Johnson et al., autologous cancellous bone grafting is known to be helpful in large defects, especially to restore the convex geometry of the articular surface [37]. Additionally, an autologous cancellous bone graft contains numerous pluripotent progenitor cells, which are also part of the cartilage restoration process in the AMIC procedure. Homburg et al. confirmed good mid-term results of AMIC in combination with cancellous bone grafting [38]. The short- and long-term results in this group are similar to those of pure chondral defects.

Further limitations need to be mentioned. First, this was a mono-center study; thus, it could be argued that external validity is limited. We are aware that no single study is capable of providing full external validity because it has been reported that large variation exists across and within countries in terms of orthopedic treatments [39]. Second, the study has a retrospective design and suffers from methodological weaknesses common in this design. Third, the data are based on patient-reported outcome measures and revision rates, but these have been shown to be important assessments for measuring patient satisfaction.

The presented long-term data emphasize that a combined strategy, meaning correct recognition of physiological rather than non-physiological knee function, may be advantageously instead of performing only a cartilage-stimulating procedure, such as the AMIC or any other cartilage repair procedure alone. Regarding the cartilage repair technique used, AMIC showed durable results in aligned knees compared to the reported results of simple microfracture and thus should be preferred in this context.

## Conclusion

The present data confirm the long-lasting clinical benefit of AMIC for focal cartilage lesions as long as the overall alignment of the lower limb and patellofemoral tracking are maintained. In our opinion, it is crucial to correct biomechanics of the knee in the same operation as this is the underlying problem leading to the cartilage lesion.
